# Influence of microbiota on immunity and immunotherapy for gastric and esophageal cancers

**DOI:** 10.1093/gastro/goaa014

**Published:** 2020-06-04

**Authors:** Xiaoli Zhang, Zui Pan

**Affiliations:** g1 Department of Biomedical Informatics, The Ohio State University, Columbus, OH, USA; g2 College of Nursing and Health Innovation, The University of Texas at Arlington, Arlington, TX, USA

**Keywords:** dysbiosis, tumor microenvironment, proton-pump inhibitors, antibiotics, checkpoint inhibitors, *H. pylori*

## Abstract

Gastric and esophageal cancers are multifactorial and multistage-involved malignancy. While the impact of gut microbiota on overall human health and diseases has been well documented, the influence of gastric and esophageal microbiota on gastric and esophageal cancers remains unclear. This review will discuss the reported alteration in the composition of gastric and esophageal microbiota in normal and disease conditions, and the potential role of dysbiosis in carcinogenesis and tumorigenesis. This review will also discuss how dysbiosis stimulates local and systemic immunity, which may impact on the immunotherapy for cancer.

## Introduction

Gastric cancers (GCs) and esophageal cancers (ECs) are the third and sixth leading cause of cancer-related death worldwide, respectively, especially in the developing countries such as in East and Central Asia [1–3]. Both malignancies are very aggressive, with the 5-year survival rate in patients <20% [4, 5]. While it is known that they are multifactorial and multistage-involved malignancies, their etiologies are poorly understood. Some studies suggest that naturally occurring microbiome, bacterial, viral, and environmental factors play a substantial role during gastric and esophageal tumorigenesis [6]. While it has been well recognized that the microbiota in the lower gastrointestinal tract have a significant impact on overall human health and disease, less is known about the influence of gastric and esophageal microbiota on immunobiology, pathophysiology, and even treatment response in GC and EC [7, 8]. For example, chronic *Helicobacter pylori* infection has been considered as the strongest risk factor for GC, yet it is unclear why only 3%–6% of the population infected with *H. pylori* actually develops a stomach tumor [9]. Even though the esophagus and stomach are anatomically connected, it is undefined whether the microbiota in the two organs contains a similar composition and diversity of microbiota. More importantly, it is uncertain whether unbalance in microbiota caused by medicines, such as antibiotics or proton-pump inhibitors (PPIs), contributes to GC or EC.

The immune system includes interlinked innate and adaptive arms, where cells from the innate immune system provide the first-line early immune response to products of infectious microorganisms through a complex network of cytokines, followed by a response from the adaptive immune system that develops various mechanisms to provide specific and long-term memory response [10]. Normally, human microbiome does not cause a pro-inflammatory response but, when the mechanisms of defense developed by the immune system are impaired or new bacteria are introduced into the system, such as the translocation of commensal bacteria through the mucosa, or under immunodeficiency, the immune system may react to the microbiome and some of the responses may trigger or facilitate tumor growth [11]. Recent discovery and development of the immunotherapy agents, specifically the checkpoint inhibitors, such as inhibitors against programmed cell death protein 1 (PD-1), programmed death-ligand 1 (PD-L1) or cytotoxic T-lymphocyte-associated protein 4 (CTLA-4) that can restore T-cell function to destroy tumor cells, have been proposed as promising options for the targeted treatment of GC or EC [12–14]. A specific subgroup of patients responding to the immunotherapy may need to be better identified.

**Figure 1.**
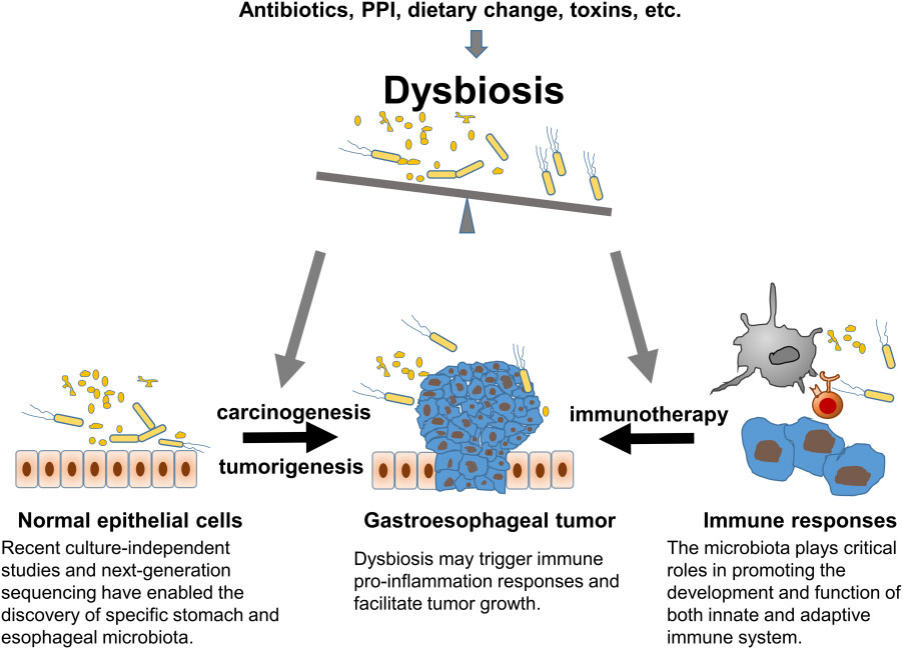
Illustration of the microbiome in gastric and esophageal cancers. The gastroesophageal microbiota plays critical roles in the programming of innate and adaptive immune responses. Imbalance of microbiota has been associated with gastric and esophageal cancers.

Therefore, this review will discuss the composition of gastric and esophageal microbiota and their potential role in carcinogenesis and tumorigenesis, as well as their effects on local and systemic immunity that will affect the results of immunotherapy for cancer (Figure 1).

## Gastric microbiota and GC

The human microbiome is essential to normal physiology because the enormous quantity of molecules produced by the microbiota can interact with the host to provide a natural defense against the colonization of pathogens [11]. The relationship between microbiota and cancer etiology has greatly intrigued biomedical researchers since the partial success of William Coley by local injection of bacteria to treat sarcomas [6]. A number of oncogenic viruses, bacteria, and helminthes have been identified and targeted by appropriate antibiotics to prevent and abort cancer, and examples include papilloma viruses for cervical carcinoma, bacteria *H. pylori* for non-cardia gastric carcinoma, and *Schistosoma hematobium* for bladder cancer [6]. Helicobacter species are present in the gastrointestinal tracts of many mammals, including human, and are considered a risk factor for GC. Since GC is a multifactorial disease, the pathophysiological stages of GC from the tumor initiation, progression, to metastasis are all indispensable for the alterations in the tumor microenvironment; consequently, gastric microbiota has attracted increasing attention, which is an important part of the tumor microenvironment [15]. GC is classified into cardia and non-cardia types according to the anatomic origin of the cancer [16]. It has been considered that increased chronic colonization of *H. pylori* can increase the risk of non-cardia cancer [17]. However, the relationship between *H. pylori* infection and gastric cardia cancer varies by populations. Two distinct etiologies of cardia-cancer subtypes were identified: one subtype is associated with gastroesophageal reflux disease (GERD), which mainly happens in patients without *H. pylori* infection; and another subtype is associated with chronic atrophic gastritis caused by *H. pylori* infection and thus presents a positive association with *H. pylori* resembling gastric non-cardia cancer[18].

Due to the fact that only 3%–6% of *H. pylori*-infected subjects developed GC within a decade [9] and that progression to GC in some subjects occurs even after the eradication of the bacterium [19], it is likely that the gastric microbiome and environmental factors contribute to the progression of disease as well. The human microbiome is composed of organisms belonging to different domains such as bacteria, archaea, eukarya, and their viruses, among which bacteria are the major inhabitants [20]. About 70% of the human microbiome is composed of bacteria that cannot be cultivated by current traditional culture-based microbiological methods [11]. Traditional culture-based studies previously suggested that the stomach was sterile in normal subjects because of its hostile acid gastric environment for microbiome colonization [7, 21]. However, emerging data have shown a large rich diversity of the bacterial population in the stomach [22] and the association of its dynamic composition with different disease states from chronic gastritis, to intestinal metaplasia, and to GC [23–26]. The recent advancements in next-generation genomic sequencing and related computational methods for phylogenetic analysis have uncovered an extremely abundant and complex microbiota in human gastrointestinal tract and uncovered their key role in metabolism, inflammation, and cancer progression [8, 27, 28]. The predominant microbiota in human stomach belongs to five phyla, including *Proteobacteria*, *Firmicutes*, *Bacteroidetes*, *Actinobacteria*, and *Fusobacteria* [29]. Based on shotgun 16S rRNA sequencing or other quantitative methods such as microarray and next-generation sequencing, studies from different groups have identified *H. pylori* as the most abundant species in GC-tumor samples [23, 30–33]. Compared to patients with chronic gastritis, the total bacteria load was relatively higher and positively correlated with *H. pylori* quantity, and the structure of tumor microbiota was more diversified in GC patients [34]. Interestingly, the community composition of the gastric microbiota was found to be significantly more diverse after surgery, with microbiota shift at the phylum level with decreased *Proteobacteria* and *Actinobacteria* and increased *Firmicutes* and *Bacteroidetes* after surgery [32]. By studying GC patients from China and Mexico, Yu *et al.* [33] showed that *H. pylori* was the most abundant member of gastric microbiota, followed by oral-associated bacteria, regardless of the *H. pylori* colonization status and the stomach anatomic sites; however, the gastric-microbiota composition did differ between paired non-malignant and tumor tissues for both Chinese and Mexican patient samples. Lin *et al.* [15] reported that GC-specific stomach microhabitats instead of GC stages or types determine the composition and diversity of the gastric microbiota. Decreased bacterial richness in peritumoral and tumoral tissues compared to normal were noted with significantly decreased *H. pylori*, *Prevotella copri*, and *Bacteroides uniformis*, and increased *Prevotella melaninogenica*, *Streptococcus anginosus*, and *Propionibacterium acnes* in the tumoral microhabitat [15]. Ferreira *et al.* [35] also reported decreased richness and diversity of microbiota in GC patients characterized by decreased *H. pylori* abundance and enrichment of other bacterial genera. Higher *H. pylori* colonization was found to influence the overall structure of the gastric microbiota in both normal and peritumoral microhabitats [15]. Another case–control study with Korean population identified *H. pylori*, *P. acnes*, and *P. copri* as strong risk factors, whereas *Lactococcus lactis* has a protective factor for GC development [36]. It seems that gastric dysbiosis with decreased gastric-microbiota richness and diversity in GC tumors play potential roles during GC tumorigenesis and tumor development, and the identification of specific species contributing to disease progression has become a pivotal task.

A major goal of microbiota studies is to identify the bacterial species responsible for promoting pathogenic changes that might contribute to carcinogenesis in the stomach. It has been recognized that *H. pylori*-induced chronic gastritis is the major risk factor for the development of GC, but the mechanism underlying how *H. pylori* and its associated microbiota promote epithelial atrophy, dysplasia, and eventual cancer is unclear. Lofgren *et al.* [37] proposed that gastric atrophy and hypochlorihydria induced by *H. pylori* infection renders the stomach more susceptible to bacterial overgrowth (gastric sysbiosis), which may subsequently lead to bacterial conversion of dietary nitrates into carcinogens. In both gastric samples and the serum of mice with *H. pylori*-associated GC, there are increased levels of IL-1, IL-17, and TNF-α, characterized as an enhanced Th17 response [38]. In addition, *H. pylori* infection was found to be associated with the lymphoid hyperplasia of gastric mucosa, which represents a preneoplastic condition of the mucosa-associated lymphoid tissues. In this case, macrophages and Th lymphocytes seem to play a key role during the anti-*H. pylori* immune response [39].

As the most abundant species in the human stomach, *H. pylori* appears to have positivistic effects on gastric microbiota, although it has not been well defined and a limited number of studies have reported contrary results. Bik *et al.* [22] found that there was no effect of *H. pylori* positivity on the composition of human gastric microbiota, which is similar to the report from Yu *et al.* [33]. However, Maldonado-Conteras *et al.* [25] reported that the microbial community differs in *H. pylori*-positive and -negative individuals, characterized by an increase in the counts of non-*Helicobacter Proteobacteria*, *Spirochaetes*, and *Acidobacteria*, and an relative decrease in the counts of *Firmicutes*, *Bacteroidetes*, and *Actinobacteria* in *H. pylori*-positive individuals compared to negative individuals. A recent study reported similar results that *H. pylori* serological status had a significant impact on gastric-microbiome diversity and composition, where *H. pylori*-positive GC patients had increased species richness and phylogenetic diversity [40]. The different results from those studies could be due to the differences used for bacterial DNA detection or differences in study populations.

## Esophageal microbiota and EC

Based upon different etiological and pathological characteristics, EC has two main types, i.e. esophageal squamous cell carcinoma (ESCC) and esophageal adenocarcinoma (EAC). While EAC is more prevalent in the USA, ESCC predominates among Asian and male African Americans [41, 42]. The use of tobacco products, including smoking and chewing tobacco, is a major risk factor for developing both ESCC and EAC [43]. Other risk factors include alcohol consumption [44], dietary zinc deficiency [45], and mechanical insults for ESCC [46] or obesity, GERD and Barrett’s esophagus (BE) for EAC [47]. Additionally, esophageal microbiota is likely associated with both ESCC and EAC.

The esophagus was initially believed to be a microbe-free site, except for transient exposure of the oral or gastric microbiota through swallowing or gastroesophageal reflux, respectively. But recent advances in culture-independent studies and next-generation sequencing have enabled the discovery of distinct esophageal microbiota, which are quite different from oral or stomach microbiota [48–52]. Pei *et al.* [49] reported that a normal esophagus is predominantly colonized by *Streptococcus* and contains five other distinct phyla, including *Firmicutes*, *Bacteroides*, *Actinobacteria*, *Proteobacteria*, and *Fusobacteria*. Alterations in the microbiome have been associated with esophageal diseases. Compared with normal esophageal microbiota, gram-negative bacteria including *Bacteroides*, *Proteobacteria*, *Fusobacteria*, and *Spirochaetes* are increased and *Streptococcus* is reduced in esophagitis and BE [53, 54]. Similarly, both ESCC and EAC are associated with more gram-negative microbiota. Zaidi *et al.* [55] reported that *E. coli* was found in only BE and EAC, but not in normal esophagus, and activation of the toll-like receptor signaling pathway is likely the mechanism underlying the associations. Higher abundance of Clostridiales and Erysipelotrichales Orders in *Firmicutes phylum* in gastric microbiota has been associated with ESCC [56]. A recent study further revealed that ESCC tumor tissues contained more *Fusobacterium* (3.2% vs 1.3%) and less *Streptococcus* (12.0% vs 30.2%) than non-tumor tissues [57].

Interestingly, *H. pylori* appears to be inversely associated with EC, especially with EAC [58]. *Helicobacter pylori* infection can impede parietal cells from secreting hydrochloric acid and thus prevent too acidic conditions (lower pH) in the gastric track. Higher pH can alleviate GERD, the leading cause of premalignant BE; thus, it is believed that *H. pylori* eventually can result in a reduction in EAC. However, the relationship between *H. pylori* infection and EC remains controversial [59, 60]. An earlier meta-analysis including 19 studies showed that *H. pylori* has a ‘beneficial’ effect on EAC with the summary odds ratio (OR) (95% confidence interval (CI)) as 0.56 (0.46–0.68) [59]. They further revealed that colonization with cytotoxin-associated gene A (CagA)-positive strains but not CagA-negative strains was inversely associated with EAC risk. Recently, Gao *et al.* [60] also performed meta-analysis including 35 studies with 345,886 patients enrolled. This analysis showed no significant correlation between *H. pylori* infection and ESCC in the general population. Instead, they only found a significant association between *H. pylori* infection and ESCC from the studies conducted in the Middle East (OR: 0.34; 95% CI: 0.22–0.52/95% CI: 0.26–0.44). They did not find any significant difference in the prevalence of *H. pylori* between the case group and the control group in EAC (8.87% vs 9.67%).

## Association between exposure of antibiotics or PPIS and increased risk of EC and GC

Given dysbiosis is associated with cancer risk in the esophagus and stomach, it is believed that antibiotics or PPIs alter the esophageal and gastric microbiota and thus may contribute to EC and GC. However, there are conflicting reports regarding the association between exposure and antibiotics or PPIs with increased risk of EC or GC. A UK-data of epidemiologic case–control study demonstrated an increased cancer risk with recurrent antibiotic exposure in specific organ sites, such as the esophagus and stomach [61], whereas another UK-data nested case–control study showed no association between the use of tetracycline and risk of gastroesophageal cancer [62]. Clearly, the definite association between antibiotics and gastroesophageal cancers requires further investigation. More importantly, future studies regarding the mechanism underlying how antibiotics alter the esophageal and gastric microbiota and how various microbes impact gastroesophageal integrity, inflammation, and carcinogenesis are warranted. Such gained knowledge could shed light on a new treatment strategy and prevention for GC and EC.

PPIs are the most commonly prescribed class of medication for the treatment of a variety of acid-related gastrointestinal disorders. PPIs include many structurally and chemically similar drugs, such as omeprazole, lansoprazole, rabeprazole, and pantoprazole. The mechanism of action is blocking the site of acid production in the parietal cell of the stomach. PPIs have been approved as rather safe to use, yet the potential association between long-term usage of PPIs with increased cancer risks remains a concern [63]. One possible mechanism underlying the association is due to PPI-induced dysbiosis as a consequence of significant alteration in the gastroesophageal environment from a low to high pH. Karmeli *et al.* [64] reported that omeprazole at the conventional dose could induce gastric dysbiosis. There is increased culture of alpha-hemolytic *streptococci*, *corynebacteria*, and *candida* species in gastric fluids. The observation was further confirmed using 16S rRNA sequencing in esophagitis and BE patients after usage of PPIs [65]. In addition to gastric microbial, this study also demonstrated that PPIs treatment has dramatic effects on esophageal microbial communities. A recent study conducted in Australian using 16S and 18S rRNA sequencing as well as shotgun metagenomics showed that PPIs treatment did not significantly affect the alpha diversity measures or global taxonomic composition of the esophageal microbiota [66]. But PPIs treatment may result in a higher number of individual bacterial taxa in GERD patients as compared with subjects with a normal esophagus. While more studies show that PPIs treatment causes altered gastroesophageal microbiota, the direct evidence and underlying mechanism of PPIs treatment involving gastroesophageal carcinogenesis certainly require further investigation.

## Gastric and esophageal microbiota and local immunity

The high number and immense diversity of microbiota within the stomach and esophagus can influence metabolism, tissue development, inflammation, and immunity [67, 68]. In a physiological state, there is a perfect balance between microbiota and the immune system at esophageal or stomach epithelia; however, a breakdown of the physiological balance in microbial composition, called ‘dysbiosis’, can cause various pathological conditions. In fact, dysbiosis has been considered a common effector in different pathogenetic pathways involved in different human diseases, especially cancer [69, 70]. It can be a consequence of inflammation induced by many factors such as hormonal perturbations, dietary compounds, toxins, and antibiotics [71]. The interplay between microbiota including bacteria, viruses, parasites, and fungi and stomach mucosal immune cells is extensive and critical, and it is regulated by a complex network of cytokines that are produced by immunologically active cells [72]. When the balance between the microbiome and immune system is impaired such as in the case of esophageal/gastric dysbiosis, the reaction of the immune system to prevent bacterial invasion and infection may trigger tumor growth [11]. Gastroesophageal microbial community profiling revealed that dysbiosis is associated with EC/GC or precancerous lesions [35, 61].

The immune system includes the interlinked innate and adaptive immune system, where innate immune defense is the first barrier that comes into play immediately after an antigen’s appearance in the body and the adaptive immune system is normally silent but it can counteract the pathogens that evade or overcome the innate immune defense [71]. An important challenge faced by the immune system is to distinguish between beneficial or pathogenic microorganisms that share similar molecular patterns such as polysaccharides or lipoproteins that can be recognized by the innate immune system. This challenge can be better tackled by the adaptive immune system, which can distinguish discrete molecular sequences and mount both pro- and anti-inflammatory responses, depending on the nature of the antigen. The innate immune system is composed primarily of immune cells such as macrophages, neutrophils, dendritic cells (DCs), and natural killer cells, and the complement system, cytokines that act to clear pathogens. The innate immune system can detect infectious agents of microorganisms and provide an important early defense. After that, DCs play a crucial role in activating the adaptive immune system, which is composed of B- and T-cells with an abundant repertoire of antigen receptors, such as Toll-like receptors (TLRs) and class II major histocompatibility complex (MHC II) molecules that are involved in pathogen sensing and antigen presentation, respectively [10, 67].

The commensal bacteria play critical roles in promoting the development and function of the adaptive immune system, and the development of the mature microbiota is regulated by host immune system components. Important effects of microbiota on the host immune system were evidenced from the study of germ-free (GF) animals that lack intestinal microbiota or are raised under GF housing conditions [67, 73]. These mice have defects in the development of both the local innate immune system such as gut-associated lymphoid tissue formation and the adaptive immune system, as well as impaired functional aspects and compromised cellular and molecular profiles of the broad immunity. Their intestinal epithelial cells show a reduced number of CD4^+^ T-cells and reduced expression of TLRs and MHC IIs [67, 73]. In addition, these mice develop abnormal spleens and lymph nodes with a decreased number of B- and T-cells in the germinal centers and parafollicular region, respectively, leading to decreased IgA and IgG levels in the serum, which, combined with gut dysbiosis, may be a cause for gluten-sensitive enteropathies in ‘common variable immunodeficiency’ (CVID) [74–76]. The impact of commensal bacterial on shaping the host immunity also showed that improper programming of the Th1/Th2 balance in GF mice towards Th2-type allergic responses can be corrected by colonization with commensal bacterial [77].

During equilibrium, host cells involved in the innate immune system respond to foreign antigens via pathogen recognition receptors such as TLRs by pathogen-associated molecular patterns (PAMPs) or microbe-associated molecular patterns (MAMPs) [78]. Therefore, circulating bacteria-derived molecules, such as lipopolysaccharide (LPS), peptidoglycan, or flagellin, which can be recognized by innate immune cells through PAMPS or MAMPs, can signal via the MyD88 (myeloid differentiation primary response protein)-dependent pathway to enhance the systemic innate immune cell response [79]. Metabolites produced by bacteria may also impact local immunity via IgA production by plasma cells to augment immunity. PAMPs act to induce the maturation of antigen-presenting cells such as DCs. Once activated, DCs can interact with and stimulate naïve T-cells to form CD4^+^ T-cells, specifically CD4^+^ T regulatory cells, and Th17 cells, and DCs may also directly stimulate CD8^+^ T-cells [80]. TLR signaling from microbial peptides to DCs and other innate immune effectors generates cytokines and interferons that act in both a paracrine and an endocrine manner at distant sites to promote systemic adaptive immunity [80]. Furthermore, upon being primed by antigen-presenting DCs, where the antigens are derived from commensal organisms, B- and T-cells, including Tregs and Th17 cells, can circulate systemically to facilitate immune responses against the same organism or similar antigens/epitopes at distant sites [81]. Dysbiosis, when the delicate balance of commensal bacteria is disrupted with the potential enrichment of pathogenic bacteria, can lead to impaired local, regional, and systemic immune responses.

As a top recognized risk factor for GC, *H. pylori* infection is an active stimulator of both the innate and adaptive immune responses. *Helicobacter pylori* colonization in the gastric mucosa triggers innate host defense leading to the expression of pro-inflammatory and anti-bacterial factors by gastric epithelial cells. This first-line defense of the gastric epithelium cells may further stimulate an innate immune response from the infiltrating cells involved in the inflammatory response, which may subsequently influence bacterial density and diversity, the level of inflammation, and eventually the generation of adaptive immune responses [82]. The severity of the inflammation and immune response is an important determinant of whether or not it leads to gastric carcinogenesis.

The inflammation and malignant transformation in esophageal and gastric epithelium may be initiated by the activation of TLRs by Gram-negative bacteria [51]. TLRs are a class of plasma membrane proteins that recognize structurally conserved molecules derived from microbes. Among several TLRs expressed in human esophageal epithelial cells, TLR4 is the most interesting one, since its natural ligand is LPS. TLR4 has been found with a higher expression in esophageal biopsy tissues removed from EAC and BE patients compared with those from normal control subjects [51]. Activation of TLR4 induces nuclear translocation of NF-κB and then the downstream target genes involved in inflammation, apoptosis blockage, innate immune responses, and adaptive immune responses, etc. Therefore, gastroesophageal dysbiosis might participate in the carcinogenesis of ECs and GCs through the LPS–TLR4–NF-κB signaling pathway.

## Microbiota and immunotherapy for GC and EC patients

Fully understanding the interactions between microbiomes and the immune system in cancer is essential for the development of precision medicine. Given the important role of microbiota in shaping host immunity, it is intuitive to understand that it could significantly influence the response and toxicity to various forms of therapeutic treatment for cancer. In human, there are two types of adaptive immune responses: one is humoral immunity mediated by antibodies from B-cells and another is cell-mediated immunity mediated by T-cells, including both CD8^+^ (Tc) and CD4^+^ (Th) cells. The discovery of proteins that can inhibit the response of T-cells provides a great opportunity for the treatment of different malignancies that show increased expression of those proteins. This negative regulation is mediated mainly by two interactions: CTLA-4 and PD-1 and its ligand PD-L1. Both CTLA-4 and PD-1 are only expressed on activated T-cells, whereas PD-1 ligands PD-L1 and PD-L2 are expressed on different cell types, with PD-L2 predominantly expressed on antigen-presenting cells and PD-L1 expressed on cell types such as innate immune cells, epithelial cells, and endothelial cells. In the tumor microenvironment, activation of both proteins protects tumor cells while CTLA-4 regulates the immune response early when the T-cells are activated, and PD-1 acts later on to induce T-cell apoptosis and eventually stop the immune response. Thus, using antibodies to directly block those negative immunological regulators (checkpoints) proved to be an important strategy for cancer immunity; particularly, the PD-1/PD-L1 inhibitors have shown signs of efficacy for different cancer treatments [10].

Immunotherapy is the treatment of disease by inducing, enhancing, or suppressing an immune response [83]. The potential use of immunotherapeutic strategies for GC and EC has received considerable attention. GC is a heterogeneous disease and it has been categorized into four subgroups based on a recent study by the Cancer Genome Atlas, including (i) tumors positive for Epstein-Barr virus (EBV), (ii) microsatellite unstable tumors (MSIs), (iii) genomically stable tumors, and (iv) tumors with chromosomal instability [84]. The EBV subgroup, which represents 15% of all GC tumors, had increased expression of CD274 and PDCD1LG2. The CD274 and PDCD1LG2 genes encode PD-L1 and PD-L2, which are immunosuppressive proteins; increased expression of these proteins indicates the presence of stable immune cells (specifically, T-cells) and supports the use of an immune checkpoint inhibitor for the treatment of those GC patients [84]. In the tumor microenvironment, the activity of T-cells is inhibited by CTLA-4 and PD-1/PD-L1, disabling the T-cells to destroy tumor cells. Combining treatment with checkpoint inhibitors can stimulate the signaling pathways that contribute to the antitumor effect of T-cells to activate the immune response. Currently, three main types of checkpoint inhibitor immunotherapy drugs, i.e. anti-CTLA-4, PD-1, and PD-L1 inhibitors, have been developed and demonstrated to be effective treatment for a variety of malignant tumor types, where the anti-PD-L1 inhibitor has been approved for advanced GC patients tested with positive PD-L1 expression [85, 86].

EC is also heterogeneous and shares similarities with GC in terms of molecular basis for immunotherapy. Only about 40% of EC patients present PD-L1 and its expression occurs predominantly on infiltrating myeloid cells but not on cancer cells [14]. In an Asian phase III trial (ONO-4538–12, ATTRACTION-2), Kang *et al.* [87] found that nivolumab, a PD-1 inhibitor, produced an improvement in overall survival vs placebo in patients with metastatic gastroesophageal cancer who had failed previous chemotherapy. Unfortunately, the majority of patients do not respond, even in patients with PD-L1^+^ tumors.

Despite the promising results for treatment with these agents, a large proportion of patients do not experience an objective response, or the response is not durable, and some patients even develop therapeutic resistance over time. Emerging evidence have shown that the gut microbiota plays a significant role in modulating responses to these immunotherapies. Initial evidence from mouse studies has demonstrated that specific microbes contribute to immune checkpoint blockade immunotherapy, including CTLA-4 and PD-1/PD-L1 blockade [88, 89]. The genus *Bifidobacterium* was found to be the key promoter for increased tumor-specific T-cell responses and increased intratumoral CD8^+^ T-cells in melanoma mice treated with anti-PD-1/PD-L1 inhibitors [88]. *Bacteroides* inoculation in GF and antibiotic-treated specific-pathogen-free mice treated with anti-CTLA-4 showed decreased tumors and a reduced incidence of colitis, which is the side effect of anti-CTLA-4 treatment, revealing that *Bacteroides* was the causation for the treatment effect, whereas mice without inoculation had markedly reduced efficacy of anti-CTLA-4 treatment [89–91]. Multiple studies have reported consensus findings that the gut microbiome affects treatment responses to checkpoint inhibitors for patients with different malignancies, such as lung cancer, kidney cancer, and melanoma [80, 92–96]. Distinct bacterial taxa were overrepresented in patients who were responders or non-responders, based on DNA sequencing analysis of stool samples before checkpoint blockade therapy, indicating an association between gut-microbiome composition and subsequent therapeutic response. Mice experiments mirroring patient data showed that mice reconstituted with fecal isolates from patients who were responders had greater benefit than mice colonized with fecal samples from non-responders with checkpoint blockade therapy [94–96], which further confirmed the association between gut microbiota and the efficacy of immunotherapy. In addition to modulating host immune effects to enhance the efficacy of immunotherapeutics, the microbiome may also play a role in reducing these treatment-associated adverse effects. As mentioned above, melanoma patients with greater bacteria abundance from the Bacteroidetes phylum were less likely to develop colitis under the treatment of anti-CTLA-4 inhibitors [90]. Furthermore, some bacterial taxa were reported to be associated with both response and toxicity or non-response/lack of response. For example, the Ruminococaceae family of the Firmicutes phylum were found to be associated with both response and toxicity, while bacterial taxa within the Bacteroidales order of the Bacteroidetes phylum were associated with a lack of response to immune checkpoint blockade (Bacteroidales), respectively [80, 90, 92–94]. In summary, these data provide evidence of the involvement of microbiota in the modulation of antitumor immune responses and responses to immune checkpoint blockade.

## Concluding remarks

The gastric and esophageal microbiota is an inherent component of host physiology that plays important roles in regulating multiple host functions. Gastroesophageal dysbiosis contributes to multiple disease occurrences, including cancer. To precisely elucidate the correlations between the microbial dynamics and pathogenesis of GC and EC, further functional and mechanistic studies are needed. In the era of big data with the establishment of reliable microbial 16S rRNA sequencing and corresponding computational skills, the study of the microbiome has brought us a completely new view of their role in modulating the human immune system and as a potentially dominant mediator in immunotherapy-based cancer treatment. However, complexities exist with regard to the optimal methods for microbiome profiling and data interpretation, along with questions regarding how other factors such as nutrients and medication affect the dynamic of the microbiome and their impact on gastroesophageal cancer treatment (Figure 1). A comprehensive understanding of the interaction between microbiome and cancers and host factors could provide the potential to optimally modulate the gastroesophageal microbiota to enhance immune-surveillance and gastric and EC therapies.

## Authors’ contributions

X.Z. and Z.P. conceptualized and drafted this manuscript. All authors read and approved the final manuscript.

## Funding

The work was supported by the Comprehensive Cancer Center Support Grant of the Ohio State University as part from the National Institutes of Health P30 CA16058-32 (to X.Z.), by R01 CA185055 from the National Cancer Institute, and S10 OD025230 from the National Heart, Lung, and Blood Institute (to Z.P.).

## Conflicts of interest

None declared.
